# Design, synthesis, and evaluation of novel pinane-based thiazolidione derivatives with anti-glioblastoma activity

**DOI:** 10.1080/14756366.2025.2553691

**Published:** 2025-09-04

**Authors:** Wei Shi, Min Hu, Jiale Han, Lei Wang, Yining Jiang, Hongmei Wu, Wei Liu, Biao Xiong, Yunyun Wang

**Affiliations:** a State Key Laboratory of Natural Medicines, Departemnt of TCMs Pharmaceuticals, School of Traditional Chinese Pharmacy, China Pharmaceutical University, Nanjing, China; b School of Pharmacy, Nantong University, Nantong, China; c Jiangsu Province Key Laboratory for Inflammation and Molecular Drug Target, Nantong University, Nantong, China; d Department of Neurosurgery, First Hospital of Jilin University, Changchun, China

**Keywords:** Pinene, thiazolidione derivatives, glioblastoma, cell apoptosis, autophagy

## Abstract

A series of pinane-based thiazolidione derivatives were synthesised, and their anti-proliferative effects were investigated by CCK-8 assay. All these compounds exhibited anti-proliferation activity against three glioblastoma cell lines (U87, T98G, and U251). Compound **C5** exhibited the strongest inhibition effect against all three cell lines. Through cellular thermal shift (CETSA) assay and drug affinity responsive target stability (DARTS) assay, compound **C5** was demonstrated to directly interact with the CDK2 protein. Additional analyses revealed that **C5** inhibited cell migration and arrested the cell cycle in glioblastoma cells while also induced mitochondrial apoptosis and autophagy. Immunoblotting analysis indicated that **C5** induced the up-regulation of Bax, cleaved caspase 3, cleaved PARP-1, P62, and LC3B, and the down-regulation of Bcl-2, caspase 3, and PARP-1. Importantly, **C5** also demonstrated efficacy in a 3D cell culture model. Together, these results highlight the potential of **C5** as a lead compound for the development of novel therapies for glioblastoma.

## Introduction

Glioblastoma (GBM) is the most prevalent cancer of the central nervous system, and it is characterised by genetic heterogeneity and infiltrative growth, resulting in high rates of recurrence, disability, and mortality^1^. The standard treatment regimen for GBM currently involves maximum surgical resection followed by radiotherapy and chemotherapy, with chemotherapy as a cornerstone of post-operative care^2^. While Temozolomide is the most widely used first-line chemotherapy agent for GBM patients^3^, its clinical effectiveness is limited because of its poor selectivity for GBM cells and its extensive resistance^2^,^4^. Hence, there is an urgent need for more effective chemotherapeutic agents.

The uncontrolled and sustained cell division has been regarded as a hallmark feature of cancer^5^. Given the pivotal role of cyclin-dependent kinases (CDKs) in regulating the cell cycle and driving proliferation, CDKs have emerged as key therapeutic targets in cancer research^6^. Among them, CDK2, a member of the serine/threonine protein kinase family, requires association with cyclin A or cyclin E to exert its kinase activity. Notably, the CDK2–cyclin E complex facilitates phosphorylation of the retinoblastoma protein (RB), thereby promoting the G1/S phase transition^7^. Similarly, the CDK2–cyclin A complex is crucial for the progression from S to G2 phase of the cell cycle^8^. Accumulating evidence indicates that CDK2 is expressed at low levels in normal tissues while it is significantly elevated in cancer cells^7^,^8^. For example, compared to the normal brain, the CDK2 expression was significantly enriched in GBM tumours. Meanwhile, CDK2 promotes hyperproliferation and is associated to poor prognosis in multiple cancer cells^9^,^10^. Therefore, CDK2 is increasingly recognised as a potential therapeutic target, prompting substantial interest in the development of CDK2 inhibitors for cancer treatment.

Medicinal plants that produce essential oils are valuable sources of potential anti-cancer agents^11^. Essential oils with a pinane core exhibit many bioactivities, and they have been widely used for the treatment of cancer, arthritis, and pain. Myrtenol, a compound with a pinane core that is isolated from natural products, exhibits a range of pharmacological properties, including antioxidant, antibacterial, antifungal, antidiabetic, anxiolytic, and gastroprotective activities^12–14^. In addition, myrtenol has been reported to exhibit antiproliferative effects on malignant cells^15^. However, despite its current widespread use in the spice industry, research on the pharmaceutical applications of myrtenol has so far been limited^16^. Thus, determining the pharmacological effects of myrtenol and its derivatives is a potential research hotspot.

In medicinal chemistry, nitrogen-containing heterocycles are considered privileged motifs owing to their broad applications in pharmaceutical chemistry. Thiazolidione derivatives, as heterocyclic compounds, play a crucial role in various bioactive natural products. Thiazolidiones contain several functional moieties, including C = O, -NH-, and -S- groups, which can interact with biomolecules at multiple sites through non-covalent bonds (e.g., π-π stacking and hydrogen bonding) to enhance their water solubility^17^,^18^. Several studies have shown that thiazolidine segments that incorporate both electron-accepting groups and electron-donating (-NH- and -S-) groups readily interact with enzymes and other receptors, including protein-tyrosine phosphatase 1B^19^, monoamine oxidases^20^, tyrosine phosphatase SHP-2^21^, α-glucosidase^22^, and cyclooxygenase-2^23^, through weak bonds such as π–π stacking, hydrogen bonds, hydrophobic effects, and van der Waals forces. Recent studies have reported that the thiazolthione scaffold exhibits inhibitory activity against CDK2^24^,^25^. Hence, incorporation of the thiazolidione moiety appears to significantly improve drug binding affinity to a range of biomolecules. Because the Schiff base, a classical pharmacophore, is generally considered advantageous for enhancing the properties of whole molecules^26^,^27^, thiazolidine derivatives modified with Schiff base can be expected to exhibit significantly enhanced anticancer activities.

**Figure 1.**
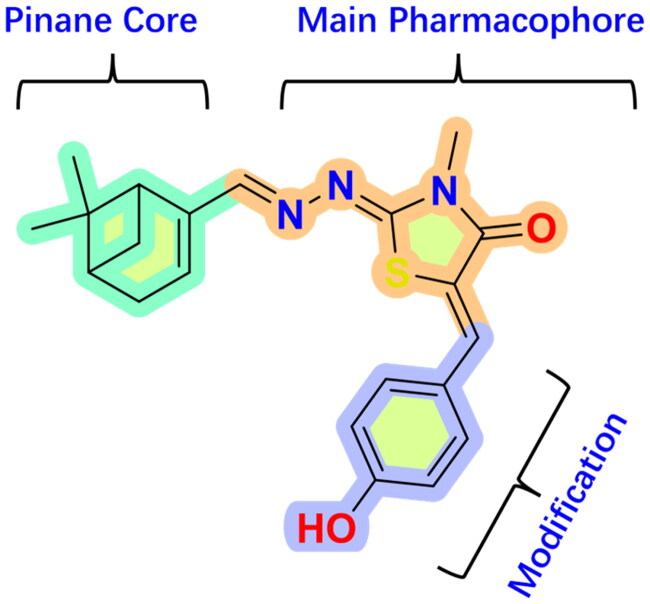
Rational design of pinane-based thiazolidine derivatives.

Considering all the aforementioned factors, our initial objective was to synthesise novel pinane derivatives incorporating the thiazolidione moiety and to evaluate their cytotoxicity against GBM cell lines (Figure 1). To further characterise the most promising compounds, we conducted a series of additional biological evaluations, including cell apoptosis assay, cell cycle analysis, and assessments of mitochondrial dysfunction, reactive oxygen species (ROS) accumulation, and the migratory capacity of U251 cells. Target identification studies were performed using CESTA, DARTS, and molecular docking techniques to confirm whether CDK2 is a potential target. Finally, to better simulate an *in vivo* environment, we employed 3D cell culture models to assess the anti-tumour efficacy of the compounds.

## Results and discussion

### General method

All reagents and solvents were purchased commercially and used without further purification unless noted otherwise. Distilled water was used for all experiments. All reactions were monitored by thin layer chromatography (TLC) and visualised under UV light at 254 nm. The column chromatography was performed on silica gel (100–200 and 200–300 mesh, Qingdao Ocean Chemical Ltd., China). The Optimelt-MPA100 melting point apparatus was used to determine melting points.^1^H NMR (400 MHz) and ^13^C NMR (100 MHz) spectra were recorded at 298 K on a Bruker AV-400 MHz spectrometer in the indicated solvents (CDCl_3_, DMSO-*d_6_*, Pyridine-*d_5_*, TMS as the internal standard). Chemical shifts are reported as ppm and coupling constants (*J*) are given in Hertz (Hz). The purities of all synthesised compounds were analysed by a Waters ACQUITY UPLC instrument (column: the ACQUITY UPLC BEH C18 column (2.1 × 100 mm, 1.7 μm); flow rate: 0.3 ml/min; detection: 254 nm; eluent A: water and 0.1% formic acid; eluent B: acetonitrile; isocratic elution: acetonitrile/water = 85:15). The purities of all tested compounds were confirmed to be more than 95% by means of UPLC methods.

### Chemistry

#### Design and synthesis of thiazolidione derivatives

**Scheme 1.**
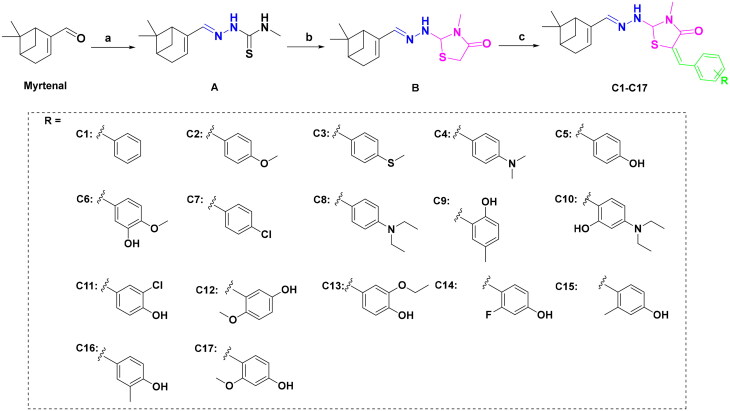
Synthesis route of probes **C1–C17** Reagents and conditions: (a) 4-methylthiosemicarbazone, HCl, EtOH, reflux, 2 h; (b) chloroacetic acid, sodium acetate, EtOH, overnight; (c) benzaldehyde derivatives, morpholine, EtOH, 60 °C, 2 h.

Compounds **C1–C17** were synthesised in three steps (as outlined in Scheme 1). Briefly, the thiosemicarbazone **A** was synthesised through a condensation reaction between the myrtenal and 4-methyl-3-thiosemicarbazide in the presence of hydrochloric acid. Next, cyclisation of compound **A** with chloroacetic acid yielded the key intermediate thiazolidinone **B.** This intermediate was subsequently reacted with substituted benzaldehyde to prepare the targeted compounds **C1–C17**. The synthesis of all new compounds is detailed in the experimental section. Each compound was thoroughly characterised using ^1^H NMR and ^13^C NMR spectroscopy.

#### Biological evaluations

##### In vitro cytotoxicity

Biological evaluation of the newly synthesised compounds (**C1–C17**) was conducted against GBM cell lines (U87, T98G, and U251). The anti-glioblastoma drug, temozolomide^7^, was used as a reference. As shown in Table 1, most of the compounds were effective against GBM cells at concentrations below 10 µM. However, the compounds containing electron-donating groups exhibited comparatively high anti-proliferative activity compared with those having containing electron-withdrawing groups. Remarkably, the *p*-hydroxyphenyl substituent compound **C5** significantly inhibited the growth of all three GBM cell lines, with IC_50_ values in the 3.12–4.25 μM range. In addition, compounds with a hydroxy group in the benzene ring exhibited stronger inhibitory effects compared to their non-hydroxylated counterparts (compounds **C6**, **C9–C13**). Hence, the introduction of a hydroxy substituent group into the benzene ring at the para- or meta-position substantially enhanced anti-cancer activity. In contrast, the introduction of methoxy, methyl, chlorine, or fluorine groups at these sites reduced the effects of the corresponding drugs on cancer cells. We speculate that the introduced hydroxyl group enhanced the hydrophilicity of the compound, thereby improving its solubility. By improving solubility, compound bioavailability is enhanced, enabling more efficient cellular uptake and distribution. This, in turn, may increase its effectiveness in inhibiting tumour growth. In summary, the presence of a hydroxyl group facilitated an increase in the antitumor activity of the corresponding compound.

**Table 1.** *In vitro* cytotoxicity data for **C1–C17** against three GBM cell lines and normal human cell line.

Compounds		IC_50_ (μM)^a^			
		U251	T98G	U87	LO2
**C1**	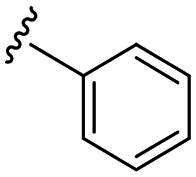	12.10 ± 0.31	9.930 ± 0.11	13.51 ± 0.43	>50
**C2**	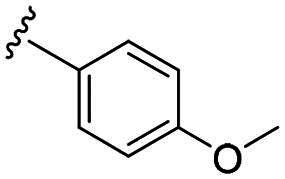	14.02 ± 0.71	10.11 ± 0.80	11.22 ± 0.26	>50
**C3**	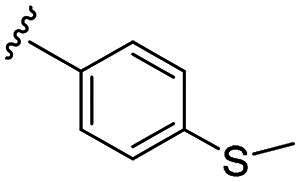	15.24 ± 0.32	16.71 ± 0.67	12.33 ± 0.65	>50
**C4**	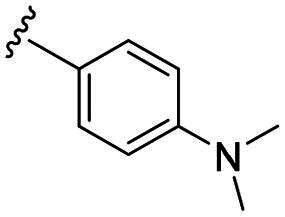	9.70 ± 0.64	8.80 ± 0.31	7.56 ± 0.76	>50
**C5**	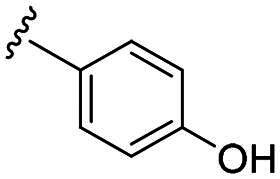	3.12 ± 0.45	4.25 ± 0.38	3.98 ± 0.34	43.2 ± 0.21
**C6**	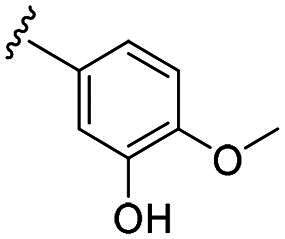	10.17 ± 0.65	7.50 ± 0.98	9.37 ± 0.43	>50
**C7**	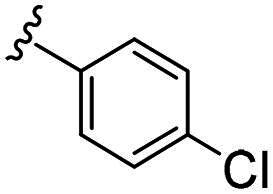	36.78 ± 0.43	33.44 ± 0.77	>50	>50
**C8**	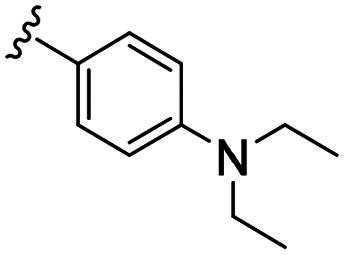	27.23 ± 0.62	38.13 ± 0.78	42.18 ± 0.46	>50
**C9**	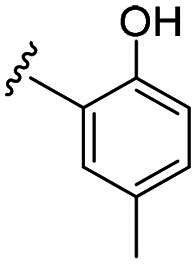	36.70 ± 0.32	32.11 ± 0.21	34.10 ± 0.57	>50
**C10**	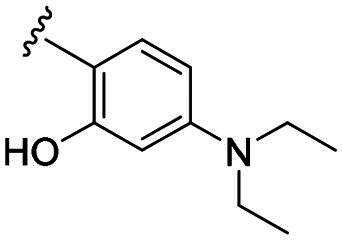	8.29 ± 0.78	8.69 ± 0.17	9.86 ± 0.09	>50
**C11**	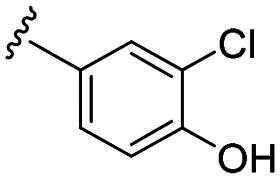	14.80 ± 0.10	11.97 ± 0.15	13.58 ± 0.21	>50
**C12**	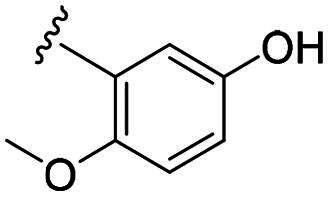	8.01 ± 0.79	7.10 ± 0.51	9.37 ± 0.35	>50
**C13**	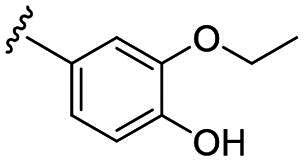	6.65 ± 0.75	5.54 ± 0.98	5.71 ± 0.23	>50
**C14**	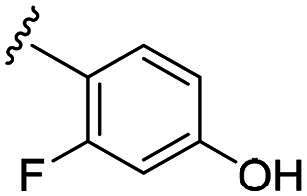	11.64 ± 0.89	14.75 ± 0.34	14.37 ± 0.73	>50
**C15**	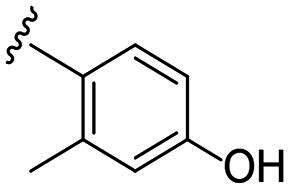	9.01 ± 0.34	7.63 ± 0.65	9.12 ± 0.43	>50
**C16**	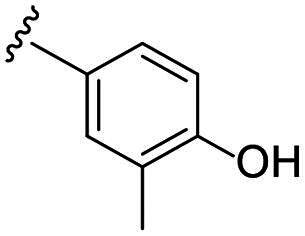	7.14 ± 0.11	6.629 ± 0.32	5.83 ± 0.56	>50
**C17**	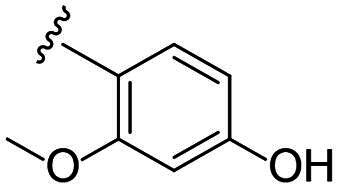	6.23 ± 0.41	5.10 ± 0.26	9.18 ± 0.20	>50

^a^ IC_50_ values are reported as the mean ± SD of three independent experiments.

##### C5-induced apoptosis and cell cycle arrest in U251 cells

**Figure 2.**
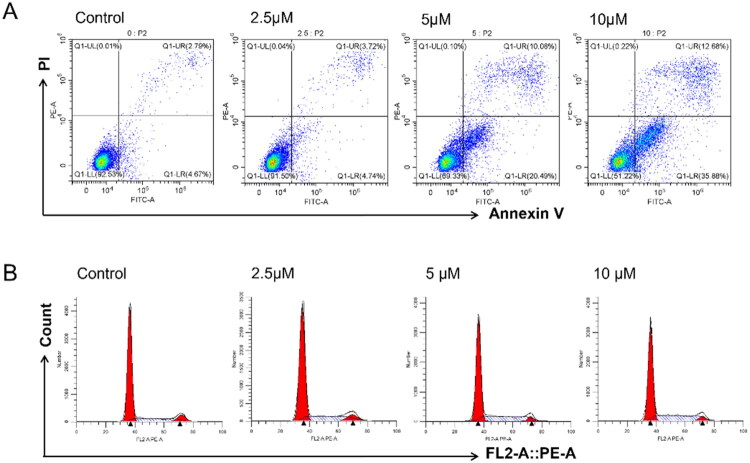
(A) Representative flow cytometry images showing the levels of Annexin V and PI in double labelled U251 glioblastoma cells after treatment with **C5** (0, 2.5, 5 and 10 µM) for 48 h. (B) Cell cycle arrest of U251 cells in the S phase after treatment with **C5** (2.5, 5, and 10 μM) for 48 h.

To explore the mechanistic basis of the inhibitory effects of **C5**, we analysed the apoptosis rates and cell cycle distribution of U251 cells treated with different doses of the compound. As shown in Figure 2(A), **C5** significantly increased the percentage of apoptotic cells in a dose-dependent manner after 48 h of treatment, and the apoptosis rate was 48.56% at the high dose of 10 μM. In addition, cells treated with **C5** for 48 h showed a decrease in the G2/M population, with a concomitant increase in the S phase population (Figure 2B). Taken together, these results suggested that **C5** inhibited the growth of GBM cells by inducing cell cycle arrest at the S phase and subsequent apoptosis.

##### Identification of CDK2 as a direct target of C5

**Figure 3.**
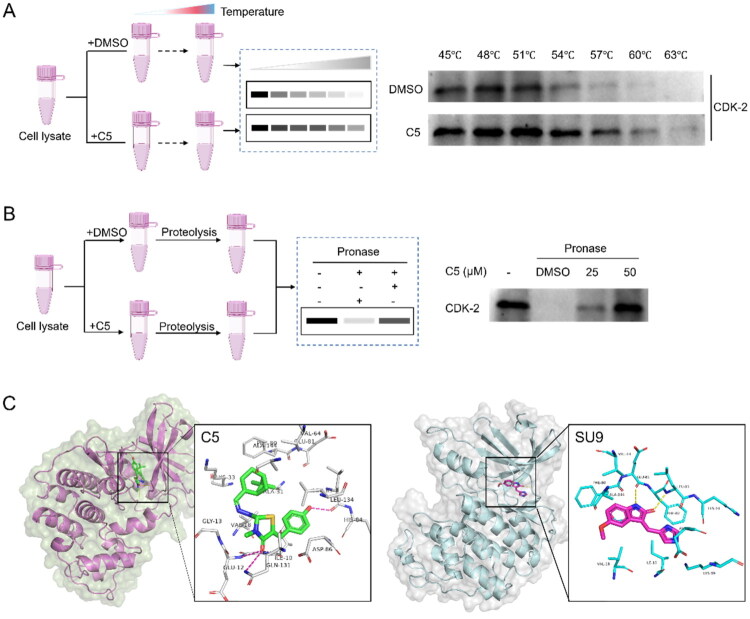
(A) CETSA assay to evaluate the binding between **C5** and CDK2; (B) DARTS assay to evaluate the binding between **C5** and CDK2 (PDB: 3PY1); (C) Docking analysis of **C5** and **SU9** with CDK2 (PDB: 3PY1).

CETSA (cellular thermal shift assay)^28^ and DARTS (drug affinity responsive target stability)^29^ are label-free target engagement assays that have been developed for use with label-free, unmodified compounds. To investigate whether compound **C5** binds directly to CDK2, we performed CETSA assay and DARTS assay for evidence of direct interaction between CDK2 and compound **C5** in cells. As shown in Figure 3(A), the thermal stability of CDK2 was substantially increased (compared to that of the control group) when U251 cells were incubated with compound **C5**, indicating that compound **C5** interacted with CDK2 directly. The results of the DARTS assay provided additional evidence that **C5** could interact with CDK2. While CDK2 stability was significantly reduced following a 30-min exposure to pronase, CDK2 pre-treated with **C5** exhibited enhanced stability. Importantly, the dose-dependent reduction in CDK2 digestion by pronase observed in the presence of **C5** was greater than that observed after DMSO pre-treatment (Figure 3B). Together, the DARTS and CETSA results indicated that CDK2 protein stability was enhanced through direct ligand-receptor interactions, consistent with the specific binding of **C5** to CDK2.

The above results suggest that compound **C5** directly binds CDK2 protein, to further investigate the potential interaction between compound **C5** and CDK2 protein, we conducted molecular docking analysis to explore possible binding modes (PDB ID: 3YP1). The docking results revealed a preferred binding mode with several prominent interactions: hydrogen bonds between the C = O atom of the thiazolidinone ring with Glu12, Gln131; a hydrogen bond between the hydroxyl group on the benzofuran ring of **C5** and Leu83; hydrophobic interactions between the pinanyl group of **C5** and amino acid residues Ile10, Phe80, and Phe82 (Figure 3C). Based on these findings, we propose that **C5** can bind the corresponding domain of CDK2.

##### C5 inhibited cell invasion and induced ROS accumulation and mitochondrial dysfunction in U251 cells

**Figure 4.**
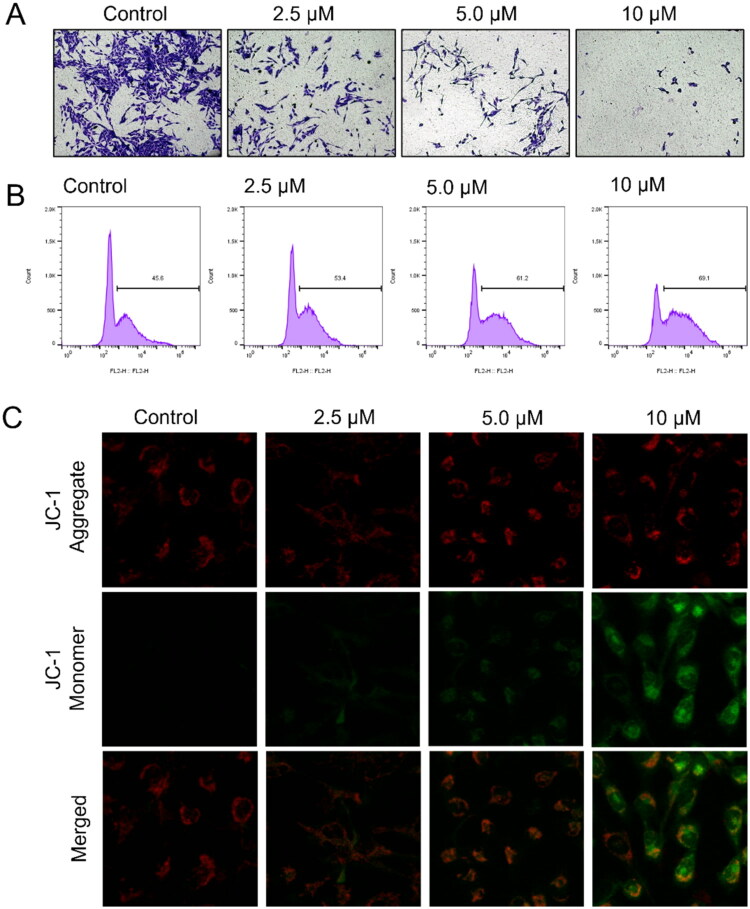
(A) Representative images showing the invasive ability of U251 cells through Matrigel-coated Transwell chambers following 24 h treatment with **C5** (0, 2.5, 5 and 10 µM) (20× magnification). (B) ROS levels in U251 cells treated with **C5** for 24 h. (C) Representative fluorescence images of U251 cells stained with JC-1 after treatment with **C5** for 24 h.

A critical factor in GBM cell invasion and migration is the ability of the tumour cells to degrade the extracellular matrix and penetrate the basement membrane^30^,^31^. To assess the effects of compound **C5** on U251 cell invasion activity, we performed a Transwell assay. As shown in Figure 4(A), treatment with **C5** for 24 h significantly decreased the number of viable cells that invaded through the Matrigel, and this effect was dose-dependent. These findings provide evidence that **C5** may inhibit GBM progression by limiting the invasion and migration of tumour cells.

High intracellular levels of ROS can cause damage to proteins, nucleic acids, lipids, membranes, and organelles, potentially triggering cell death processes such as apoptosis. While low to moderate levels of ROS are essential for regulating normal physiological functions, including cell cycle progression, proliferation, differentiation, migration, and cell death, elevated ROS levels are known to cause cellular damage^32^,^33^. To evaluate the effects of compound **C5** on intracellular ROS accumulation, the flow cytometric analysis was performed. Cells were treated with varying concentrations of **C5** (0, 2.5, 5, and 10 μM) in the presence of 2′,7′-dichlorofluorescein diacetate (DCFH-DA). As shown in Figure 4(B), incubation with 5 μM of compound **C5** significantly increased intracellular ROS levels. Hence, **C5** can significantly increase intracellular ROS levels in U251 cells and may contribute to their apoptosis.

Mitochondria are significant mediators of cell metabolism, and they are the producers and targets of ROS^34^. Given the observed accumulation of intracellular ROS in **C5**-treated U251 cells, we extended our investigation to assess the impact of **C5** on mitochondrial membrane potential (MMP). To assess this, we utilised the JC-1 fluorescent probe to monitor MMP in U251 cells treated with compound **C5**. As shown in Figure 4(C), a dose-dependent decrease in red fluorescence (JC-1 aggregates) and a corresponding enhancement of green fluorescence (JC-1 monomer) were observed by fluorescence microscopy after treatment with increasing concentrations of **C5**. These findings suggest that compound **C5** can disrupt mitochondrial function by reducing the MMP in U251 cells.

##### C5 regulated apoptosis-related and autophagy-related proteins in U251 cells

**Figure 5.**
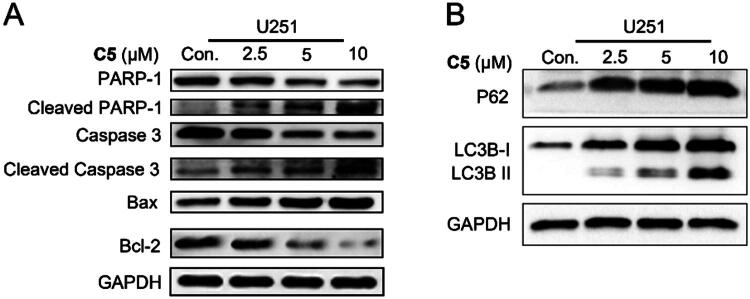
Immunoblotting analysis showing expression of PARP-1, cleaved PARP-1, caspase 3, cleaved caspase 3, Bax, and Bcl-2 in U251 cells after **C5** treatment for 24 h. GAPDH was used as the loading control.

The above experiments indicated that compound **C5** can increase reactive oxygen species (ROS) levels, eventually leading to mitochondrial damage. While mitochondria are considered the “powerhouses” of eukaryotic cells, these essential organelles also regulate calcium homeostasis, signal transduction, and cell death^35^. Indeed, mitochondrial depolarisation is commonly regarded as an early indicator of cell apoptosis^36^,^37^. Apoptosis is a type of programmed cell death, a signalling process that is vital for preventing diseases like cancer. To determine whether **C5** induced apoptosis via the mitochondrial pathway, we analysed the expression of apoptosis-related proteins. As shown in Figure 5(A), treatment with **C5** induced in a dose-dependent downregulation of the anti-apoptotic protein Bcl-2 and upregulation of the pro-apoptotic protein Bax. Additionally, **C5** enhanced PARP cleavage and caspase-3 activation in a concentration-dependent manner.

The sensitivity of cancer cells to therapeutic compounds is also dependent on autophagy and autophagic flux^38^,^39^. Accordingly, changes in the expression levels of autophagic proteins in U251 cells after **C5** treatment were also investigated. As shown in Figure 5(B), **C5** significantly enhanced the expression of P62 and LC3B-II in U251 cells. In summary, **C5** could induce apoptosis in GBM cells via activation of caspase-3, Bcl-2, and cleavage of PARP, and it could also promote autophagy.

##### C5 decreased the clonogenicity of U251 cells

**Figure 6.**
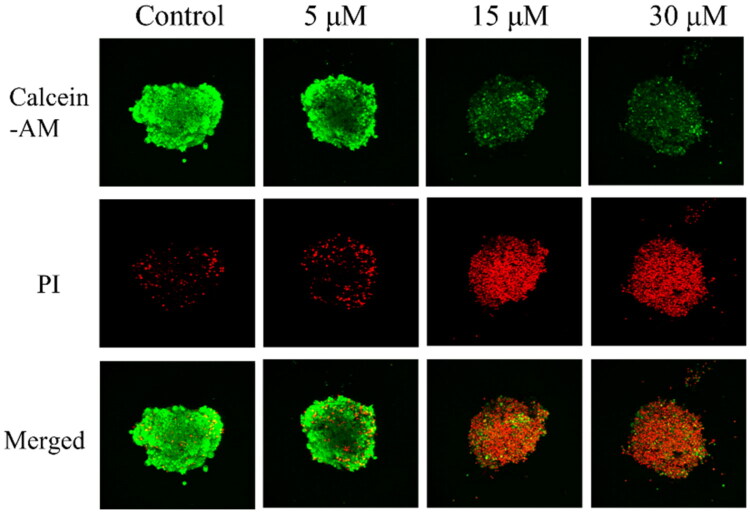
Representative images of **C5-**treated U251 cells stained with calcein-AM (green fluorescence: live) and PI (red fluorescence: dead).

Most of the anticancer drugs currently available have been developed using 2D culturing methods based on a cancer cell monolayer. However, tumour development cannot be faithfully modelled using 2D models, and the anticancer effects of drug candidates are difficult to assess accurately using 2D models^40^,^41^. Therefore, a 3D cell culture model using multicellular tumour spheroids (MCTSs) was also used to evaluate the therapeutic efficacy of **C5** in U251 cancer cells. After treatment with **C5** for 24 h, the U251 cells in MCTSs were stained with PI and Calcein AM in DMEM for 1.5 h, and the MCTSs were then observed under laser scanning confocal microscopy to analyse live/dead U251 cells (Figure 6). In untreated spheroids, most U251 cells were viable (green fluorescence), and only a few dead cells (red fluorescence) were present in the centre of sphere, possibly as a result of overgrowth and a consequent lack of nutrients and oxygen (which is known to induce apoptosis). In **C5-**treated spheroids, this necrotic core expanded with increasing concentration of **C5** until most of the U251 cells were dead (the **C5-**treated MCTSs shown below are broadly representative). Taken together, these results demonstrated that compound **C5** decreased the viability of U251 cells in a 3D model in a dose-dependent manner.

## Conclusion

In this study, we designed and synthesised a series of novel, pinane-based thiazolidione derivatives. Some compounds exhibited significant antiproliferative activities against GBM cell lines. Of these, compound **C5** demonstrated broad-spectrum antiproliferative activity against three GBM cancer cell lines with IC_50_ values < 5 μM. In addition, DARTS and CETSA experiments revealed that compound **C5** directly interacted with CDK2, providing evidence that CDK2 is a target of **C5**. Additional experiments demonstrated that compound **C5** effectively inhibited colony formation and cell migration in U251 cells, induced G2/M phase cycle arrest, and regulated the expression levels of cell cycle-related proteins. Importantly, compound **C5** was also shown to induce apoptosis by modulating apoptosis-related proteins. In summary, **C5** was demonstrated to inhibit migration, arrest the cell cycle, stimulate autophagy, and induce apoptosis (via the mitochondria apoptosis pathway) in GBM cells. The antitumor potency of **C5** was further confirmed utilising 3D cell culture. Together, the above results provide firm evidence that compound **C5** represents a novel thiazolidione CDK2 inhibitor with potent anti-glioma activity.

### Experimental section

#### Synthesis of pinane based thiazolidione derivatives

***Synthesis of compound A:*** A three-necked flask was charged with l mmol myrtenal, 1.2 mmol thiosemicarbazide, 20 ml of ethyl alcohol, and three drops of concentrated hydrochloric acid. The mixture was reacted at 50 °C for 2 h. Upon completion of the reaction, the reacted solution was cooled to room temperature, resulting in the formation of a white solid. The pure product was then separated out and isolated by recrystallization from methanol.^1^H NMR (400 MHz, CDCl_3_) *δ* 10.37 (s, 1H), 7.58 (s, 1H), 7.27 (s, 1H), 5.97 (s, 1H), 3.16 (d, *J* = 4.9 Hz, 3H), 2.82 (t, *J* = 5.6 Hz, 1H), 2.47 − 2.33 (m, 3H), 2.10 (s, 1H), 1.29 (s, 3H), 1.07 (d, *J* = 9.0 Hz, 1H), 0.73 (s, 3H). ^13^C NMR (101 MHz, CDCl_3_) *δ* 177.67, 144.67, 144.57, 134.05, 40.71, 39.94, 37.69, 32.51, 31.07, 30.93, 26.04, 20.89.

***Synthesis of compound B:*** An equimolar mixture of chloroacetic acid and compound ***A*** (2 mmol) was dissolved in 20 ml of absolute alcohol, and the mixture solution was stirred at 80 °C for 10 h. After the reaction, 20 ml of water was added, and the solution was left standing overnight. The white solid was filtered and washed with water to get compound **B**, yield 50%. ^1^H NMR (400 MHz, CDCl_3_) *δ* 8.01 (s, 1H), 6.10 (s, 1H), 3.74 (s, 2H), 3.26 (s, 3H), 3.02 (t, *J* = 6.2 Hz, 1H), 2.51–2.44 (m, 3H), 2.16 (s, 1H), 1.36 (s, 3H), 1.17 (d, *J* = 9.0 Hz, 1H), 0.81 (s, 3H). ^13^C NMR (101 MHz, CDCl_3_) *δ* 172.10, 162.88, 159.47, 146.23, 135.26, 40.72, 40.25, 37.65, 32.72, 32.39, 31.17, 29.70, 26.08, 20.92.

***Synthesis of target compounds C1- C17:*** Compound **B** and substituted benzaldehyde were dissolved in ethyl alcohol, with a catalytic amount of piperidine added. The mixture was reacted at 80 °C for 2 h. Upon completion, the reaction solution was cooled to room temperature, and the precipitated crystalline powder was filtered, washed with less ethanol, and dried to obtain the target compound **C1- C17**.

*2-(((6,6-Dimethylbicyclo[3.1.1]hept-2-en-2-yl)methylene)hydrazineylidene)-5–(4-methoxybenzylidene)-3-methylthiazolidin-4-one (****C1****):* yellow solid, ^1^H NMR (400 MHz, CDCl_3_) *δ* 8.08 (s, 1H), 7.70 (s, 1H), 7.59 (d, *J* = 7.1 Hz, 2H), 7.48 (t, *J* = 7.7 Hz, 2H), 7.39 (t, *J* = 7.4 Hz, 1H), 6.15 (s, 1H), 3.42 (s, 3H), 3.14 − 3.06 (m, 1H), 2.59 − 2.46 (m, 3H), 2.19 (s, 1H), 1.65 (s, 1H), 1.41 (s, 3H), 1.21 (d, *J* = 9.1 Hz, 1H), 0.84 (s, 3H). ^13^C NMR (101 MHz, CDCl_3_) *δ* 167.02, 160.20, 158.55, 146.28, 136.10, 134.08, 130.38, 130.10, 129.60, 129.04, 122.39, 40.78, 40.30, 37.76, 32.84, 31.26, 29.82, 26.13, 20.96. UPLC, t_R_ = 8.208 min, purity 97.94%.

*2-(((6,6-Dimethylbicyclo[3.1.1]hept-2-en-2-yl)methylene)hydrazineylidene)-5–(4-methoxybenzylidene)-3-methylthiazolidin-4-one (****C2****):* yellow solid, ^1^H NMR (400 MHz, CDCl_3_) *δ* 8.08 (s, 1H), 7.66 (s, 1H), 7.54 (d, *J* = 8.8 Hz, 2H), 7.01 (d, *J* = 8.8 Hz, 2H), 6.14 (s, 1H), 3.86 (s, 3H), 3.41 (s, 3H), 3.11 (td, J = 5.7, 1.5 Hz, 1H), 2.60 − 2.39 (m, 3H), 2.19 (s, 1H), 1.42 (s, 3H), 1.21 (d, *J* = 9.0 Hz, 1H), 0.84 (s, 3H). ^13^C NMR (101 MHz, CDCl_3_) *δ* 167.26, 160.76, 159.95, 158.85, 146.30, 135.86, 131.98, 130.30, 126.79, 119.52, 114.60, 55.44, 40.80, 40.30, 37.77, 32.82, 31.27, 29.76, 26.14, 20.96. UPLC, t_R_ = 4.455 min, purity 95.47%.

*2-(((6,6-Dimethylbicyclo[3.1.1]hept-2-en-2-yl)methylene)hydrazineylidene)-3-methyl-5–(4-(methylthio)benzylidene)thiazolidin-4-one (****C3****):* yellow solid, ^1^H NMR (400 MHz, CDCl_3_) *δ* 8.08 (s, 1H), 7.64 (s, 1H), 7.50 (d, *J* = 8.5 Hz, 2H), 7.32 (d, *J* = 8.5 Hz, 2H), 6.15 (s, 1H), 3.41 (s, 3H), 3.09 (t, *J* = 5.6 Hz, 1H), 2.59–2.46 (m, 6H), 2.19 (s, 1H), 1.64 (s, 1H), 1.42 (s, 3H), 1.21 (d, *J* = 9.1 Hz, 1H), 0.84 (s, 3H). ^13^C NMR (101 MHz, CDCl_3_) *δ* 167.09, 160.11, 158.54, 146.27, 141.53, 136.05, 130.47, 130.44, 129.87, 126.02, 121.20, 40.78, 40.30, 37.77, 32.84, 31.26, 29.82, 26.14, 20.96, 15.10. UPLC, t_R_ = 7.279 min, purity 99.18%.

*5–(4-(Dimethylamino)benzylidene)-2-(((6,6-dimethylbicyclo[3.1.1]hept-2-en-2-yl)methylene)hydrazineylidene)-3-methylthiazolidin-4-one (****C4****):* yellow solid, ^1^H NMR (400 MHz, CDCl_3_) *δ* 8.08 (s, 1H), 7.64 (s, 1H), 7.49 (d, *J* = 8.9 Hz, 2H), 6.76 (d, *J* = 9.0 Hz, 2H), 6.13 (s, 1H), 3.40 (s, 3H), 3.16 − 3.10 (m, 1H), 3.05 (s, 6H), 2.93 (d, *J* = 2.9 Hz, 1H), 2.58 − 2.46 (m, 3H), 2.19 (tdd, *J* = 6.7, 3.0, 1.6 Hz, 1H), 1.64 (s, 2H), 1.42 (s, 3H), 1.22 (d, J = 9.0 Hz, 1H), 0.84 (s, 3H). ^13^C NMR (101 MHz, CDCl_3_) *δ* 167.63, 159.46, 151.05, 146.36, 135.36, 132.17, 131.42, 121.71, 115.83, 111.97, 40.81, 40.31, 40.10, 37.78, 32.79, 31.30, 29.65, 26.16, 20.96. UPLC, t_R_ = 6.459 min, purity 97.91%.

*2-(((6,6-Dimethylbicyclo[3.1.1]hept-2-en-2-yl)methylene)hydrazineylidene)-5–(4-hydroxybenzylidene)-3-methylthiazolidin-4-one (****C5****):* yellow solid, ^1^H NMR (400 MHz, CDCl_3_) *δ* 8.09 (s, 1H), 7.65 (s, 1H), 7.48 (d, *J* = 8.7 Hz, 2H), 6.97 (d, *J* = 8.7 Hz, 2H), 6.16 (s, 1H), 3.42 (s, 3H), 3.10 (t, J = 6.3 Hz, 1H), 2.58 − 2.41 (m, 3H), 2.18 (s, 1H), 1.39 (s, 3H), 1.21 (d, J = 9.0 Hz, 1H), 0.83 (s, 3H).^13^C NMR (101 MHz, CDCl_3_) *δ* 167.51, 160.19, 158.87, 157.49, 146.21, 136.27, 132.26, 130.70, 126.60, 119.19, 116.24, 116.03, 40.77, 40.31, 37.77, 32.84, 31.27, 29.82, 26.11, 20.94. UPLC, t_R_ = 3.971 min, purity 95.15%.

*2-(((6,6-Dimethylbicyclo[3.1.1]hept-2-en-2-yl)methylene)hydrazineylidene)-5–(4-hydroxy-3-methoxybenzylidene)-3-methylthiazolidin-4-one (****C6****):* yellow solid, ^1^H NMR (400 MHz, DMSO-*d6*) *δ* 9.90 (s, 1H), 8.13 (s, 1H), 7.60 (s, 1H), 7.23 (d, *J* = 2.1 Hz, 1H), 7.10 (dd, *J* = 8.3, 2.0 Hz, 1H), 6.95 (d, J = 8.2 Hz, 1H), 6.29 (s, 1H), 3.84 (s, 3H), 3.38 (s, 1H), 3.28 (s, 3H), 3.04–2.92 (m, 1H), 2.49–2.37 (m, 2H), 2.16 (s, 1H), 1.35 (s, 3H), 1.11 (d, *J* = 8.9 Hz, 1H), 0.78 (s, 3H). ^13^C NMR (101 MHz, DMSO-*d6*) *δ* 166.64, 159.49, 159.07, 149.31, 148.29, 145.78, 136.40, 130.86, 125.47, 124.02, 118.23, 116.61, 114.80, 55.85, 37.67, 32.73, 31.11, 30.04, 26.36, 21.30. UPLC, t_R_ = 3.467 min, purity 99.55%.

*5–(4-Chlorobenzylidene)-2-(((6,6-dimethylbicyclo[3.1.1]hept-2-en-2-yl)methylene) hydrazineylidene)-3-methylthiazolidin-4-one (****C7****):* yellow solid, ^1^H NMR (400 MHz, CDCl_3_) *δ* 8.09 (s, 1H), 7.65 (s, 1H), 7.48 (d, *J* = 8.7 Hz, 2H), 6.97 (d, *J* = 8.7 Hz, 2H), 6.16 (s, 1H), 3.42 (s, 3H), 3.09 (t, *J* = 5.5 Hz, 1H), 2.58–2.46 (m, 3H), 2.18 (s, 1H), 1.39 (s, 3H), 1.21 (d, *J* = 9.0 Hz, 1H), 0.83 (s, 3H). ^13^C NMR (101 MHz, CDCl_3_) *δ* 167.51, 160.19, 158.87, 157.49, 146.21, 136.27, 132.27, 130.70, 126.60, 119.19, 116.24, 40.77, 40.31, 37.77, 32.85, 31.27, 29.83, 26.11, 20.95. UPLC, t_R_ = 7.406 min, purity 99.20%.

*5–(4-(Diethylamino)benzylidene)-2-(((6,6-dimethylbicyclo[3.1.1]hept-2-en-2-yl)methylene) hydrazineylidene)-3-methylthiazolidin-4-one (****C8****):* yellow solid, ^1^H NMR (400 MHz, Pyridine-*d5*) *δ* 10.19 (s, 2H), 9.85 (s, 1H), 9.43 (s, 1H), 9.09–8.98 (m, 3H), 8.03 (d, *J* = 9.0 Hz, 2H), 4.92 (s, 4H), 4.67 (q, *J* = 7.1 Hz, 4H), 3.81 (dt, *J* = 6.1, 3.6 Hz, 3H), 3.45 (s, 1H), 2.62 (s, 3H), 2.60 (d, *J* = 8.9 Hz, 1H), 2.47 (t, *J* = 7.0 Hz, 6H), 2.26 (s, 3H). ^13^C NMR (101 MHz, Pyridine-*d5*) *δ* 169.25, 161.79, 161.46, 151.07, 148.44, 134.74, 133.48, 123.00, 117.29, 113.86, 46.44, 42.96, 42.60, 39.72, 34.81, 33.38, 31.68, 27.92, 22.97, 14.56. UPLC, t_R_ = 6.871 min, purity 99.45%.

*2-(((6,6-Dimethylbicyclo[3.1.1]hept-2-en-2-yl)methylene)hydrazineylidene)-5–(2-hydroxy-5-methylbenzylidene)-3-methylthiazolidin-4-one (****C9****):* yellow solid, ^1^H NMR (400 MHz, DMSO-*d6*) *δ* 10.19 (s, 1H), 8.14 (s, 1H), 7.89 (s, 1H), 7.22 (s, 1H), 7.11 (d, *J* = 8.4 Hz, 1H), 6.87 (d, *J* = 8.2 Hz, 1H), 6.30 (s, 1H), 3.38 (s, 1H), 3.28 (s, 3H), 2.97 (t, *J* = 5.6 Hz, 1H), 2.44 (t, *J* = 21.0 Hz, 2H), 2.27 (s, 3H), 2.16 (s, 1H), 1.35 (s, 3H), 1.11 (d, *J* = 8.9 Hz, 1H), 0.78 (s, 3H). ^13^C NMR (101 MHz, DMSO-*d6*) *δ* 166.64, 159.71, 158.97, 155.33, 145.76, 136.53, 132.79, 128.90, 128.45, 125.45, 120.73, 120.52, 116.41, 37.68, 32.74, 31.11, 30.07, 26.34, 21.29, 20.77. UPLC, t_R_ = 5.592 min, purity 98.76%.

*5–(3-Chloro-4-hydroxybenzylidene)-2-(((6,6-dimethylbicyclo[3.1.1]hept-2-en-2-yl)methylene)hydrazineylidene)-3-methylthiazolidin-4-one (****C10****):* yellow solid, ^1^H NMR (400 MHz, CDCl_3_) *δ* 8.32 (s, 1H), 8.08 (s, 1H), 7.47 (d, *J* = 8.9 Hz, 1H), 6.36 (d, *J* = 8.9 Hz, 1H), 6.15 (d, *J* = 29.7 Hz, 2H), 3.46 − 3.31 (m, 7H), 3.12 (t, *J* = 5.5 Hz, 1H), 2.60 − 2.39 (m, 3H), 2.18 (s, 1H), 1.41 (s, 3H), 1.19 (t, *J* = 7.0 Hz, 7H), 0.84 (s, 3H). ^13^C NMR (101 MHz, CDCl_3_) *δ* 168.72, 160.13, 159.29, 158.64, 150.84, 146.35, 135.35, 130.67, 127.42, 112.91, 109.57, 104.88, 97.83, 44.60, 40.77, 40.22, 37.79, 32.79, 31.31, 29.66, 26.16, 20.98, 12.77. UPLC, t_R_ = 2.800 min, purity 98.27%.

*2-(((6,6-Dimethylbicyclo[3.1.1]hept-2-en-2-yl)methylene)hydrazineylidene)-5–(3-hydroxy-4-methoxybenzylidene)-3-methylthiazolidin-4-one (****C11****):* yellow solid, ^1^H NMR (400 MHz, DMSO-*d6*) *δ* 9.47 (s, 1H), 8.15 (s, 1H), 7.54 (s, 1H), 7.10 (d, *J* = 5.2 Hz, 3H), 6.32 (s, 1H), 3.84 (s, 3H), 3.28 (s, 3H), 3.06–2.94 (m, 1H), 2.45 (t, *J* = 18.8 Hz, 2H), 2.17 (s, 1H), 1.38 (s, 3H), 1.12 (d, *J* = 8.9 Hz, 1H), 0.79 (s, 3H). ^13^C NMR (101 MHz, DMSO-*d6*) *δ* 166.57, 160.05, 158.62, 150.02, 147.29, 145.68, 136.85, 130.58, 126.70, 123.63, 118.92, 116.30, 112.87, 56.09, 37.77, 32.74, 31.12, 30.10, 26.42, 21.32. UPLC, t_R_ = 6.710 min, purity 98.15%.

*2-(((6,6-Dimethylbicyclo[3.1.1]hept-2-en-2-yl)methylene)hydrazineylidene)-5–(3-ethoxy-4-hydroxybenzylidene)-3-methylthiazolidin-4-one (****C12****):* yellow solid, ^1^H NMR (400 MHz, DMSO-*d6*) *δ* 9.84 (s, 1H), 8.14 (s, 1H), 7.58 (s, 1H), 7.25–7.04 (m, 2H), 6.95 (d, *J* = 8.4 Hz, 1H), 6.30 (s, 1H), 4.10 (d, *J* = 7.1 Hz, 2H), 3.37 (s, 2H), 3.28 (s, 3H), 2.98 (s, 1H), 2.41 (d, *J* = 17.5 Hz, 2H), 2.17 (s, 1H), 1.37 (d, *J* = 11.4 Hz, 6H), 1.11 (d, *J* = 8.8 Hz, 1H), 0.78 (s, 3H). ^13^C NMR (101 MHz, DMSO-*d6*) *δ* 166.67, 159.40, 159.25, 149.54, 147.41, 145.79, 136.41, 130.89, 124.57, 118.22, 116.69, 115.27, 64.15, 37.68, 32.73, 31.11, 30.04, 26.39, 21.30, 15.09. UPLC, t_R_ = 3.400 min, purity 96.44%.

*2-(((6,6-Dimethylbicyclo[3.1.1]hept-2-en-2-yl)methylene)hydrazineylidene)-5–(2-hydroxy-4-methoxybenzylidene)-3-methylthiazolidin-4-one (****C13****):* yellow solid, ^1^H NMR (400 MHz, DMSO-*d6*) *δ* 10.53 (s, 1H), 8.14 (s, 1H), 7.89 (s, 1H), 7.38 (d, *J* = 8.7 Hz, 1H), 6.64 (d, *J* = 8.9 Hz, 1H), 6.51 (d, *J* = 2.6 Hz, 1H), 6.30 (s, 1H), 3.77 (s, 3H), 3.28 (s, 3H), 2.97 (t, *J* = 5.6 Hz, 1H), 2.42 (d, *J* = 19.9 Hz, 1H), 2.16 (s, 1H), 1.36 (s, 3H), 1.11 (d, *J* = 8.5 Hz, 1H), 0.78 (s, 3H). ^13^C NMR (101 MHz, DMSO-*d6*) *δ* 166.67, 159.40, 159.25, 149.54, 147.41, 145.79, 136.41, 130.89, 124.57, 118.22, 116.69, 115.27, 64.15, 37.68, 32.73, 31.11, 30.04, 26.39, 21.30, 15.09. UPLC, t_R_ = 3.751 min, purity 96.29%.

*2–(2-((6,6-Dimethylbicyclo[3.1.1]hept-2-en-2-yl)methylene)hydrazineyl)-5-((E)-2-fluoro-4-hydroxybenzylidene)-3-methylthiazolidin-4-one (****C14****):* yellow solid, ^1^H NMR (400 MHz, DMSO) *δ* 10.72 (s, 1H), 8.16 (s, 1H), 7.62 (s, 1H), 7.53 − 7.42 (m, 1H), 6.91 − 6.81 (m, 1H), 6.78 − 6.68 (m, 1H), 6.38 − 6.28 (m, 1H), 3.29 (s, 3H), 3.02 − 2.93 (m, 1H), 2.58 − 2.52 (m, 1H), 2.47 − 2.39 (m, 1H), 2.17 (s, 1H), 1.37 (s, 3H), 1.25 (d, *J* = 10.7 Hz, 1H), 1.11 (d, *J* = 8.9 Hz, 1H), 0.78 (s, 3H); ^13^C NMR (101 MHz, DMSO) *δ* 166.4, 160.3, 158.2, 145.7, 137.1, 130.5, 121.3, 113.5, 112.6, 103.8, 103.6, 43.4, 37.8, 32.8, 31.1, 30.2, 26.4, 21.3. UPLC, t_R_ = 5.002 min, purity 95.60%.

*2–(2-((6,6-Dimethylbicyclo[3.1.1]hept-2-en-2-yl)methylene)hydrazineyl)-5-((E)-4-hydroxy-2-methylbenzylidene)-3-methylthiazolidin-4-one (****C15****):* yellow solid, ^1^H NMR (400 MHz, CDCl_3_) δ 8.09 (s, 1H), 7.86 (s, 1H), 7.53 (d, *J* = 8.5 Hz, 1H), 6.88 − 6.81 (m, 1H), 6.76 (d, *J* = 2.6 Hz, 1H), 6.15 (s, 1H), 3.43 (s, 3H), 3.16 − 3.00 (m, 1H), 2.68 − 2.44 (m, 3H), 2.39 (s, 3H), 2.18 (d, *J* = 6.0 Hz, 1H), 1.37 (s, 3H), 1.19 (d, *J* = 9.0 Hz, 1H), 0.82 (s, 3H); ^13^C NMR (101 MHz, CDCl_3_) δ 167.3, 160.1, 159.2, 157.1, 146.1, 141.4, 136.3, 129.9, 128.4, 125.5, 120.5, 117.9, 113.5, 40.7, 40.2, 37.7, 32.8, 31.2, 29.8, 26.1, 20.9, 20.2. UPLC, t_R_ = 5.858 min, purity 98.69%.

*2–(2-((6,6-Dimethylbicyclo[3.1.1]hept-2-en-2-yl)methylene)hydrazineyl)-5-((E)-4-hydroxy-3-methylbenzylidene)-3-methylthiazolidin-4-one (****C16****):* yellow solid, ^1^H NMR (400 MHz, DMSO) *δ* 10.18 (s, 1H), 8.14 (s, 1H), 7.55 (s, 1H), 7.45 − 7.22 (m, 2H), 6.95 (d, *J* = 8.3 Hz, 1H), 6.31 (s, 1H), 3.28 (s, 3H), 3.06 − 2.93 (m, 1H), 2.54 (d, *J* = 5.5 Hz, 1H), 2.47 − 2.38 (m, 1H), 2.17 (s, 4H), 1.38 (s, 3H), 1.23 (s, 1H), 1.12 (d, *J* = 8.8 Hz, 1H), 0.79 (s, 3H); ^13^C NMR (101 MHz, DMSO) *δ* 166.7, 159.8, 158.8, 158.2, 145.8, 136.6, 133.8, 130.8, 129.7, 125.5, 124.9, 117.7, 115.9, 37.7, 32.8, 31.1, 30.1, 26.4, 21.3, 16.5. UPLC, t_R_ = 5.005 min, purity 98.38%.

*2–(2-((6,6-Dimethylbicyclo[3.1.1]hept-2-en-2-yl)methylene)hydrazineyl)-5-((E)-4-hydroxy-2-methoxybenzylidene)-3-methylthiazolidin-4-one (****C17****):* yellow solid, ^1^H NMR (400 MHz, CDCl_3_) *δ* 8.08 (s, 1H), 7.63 (s, 1H), 7.22 − 7.13 (m, 1H), 7.08 (d, *J* = 2.0 Hz, 1H), 7.04 (d, *J* = 8.3 Hz, 1H), 6.34 − 5.97 (m, 1H), 3.97 (s, 3H), 3.41 (s, 3H), 3.13 − 3.00 (m, 1H), 2.58 − 2.50 (m, 2H), 2.49 − 2.42 (m, 1H), 2.24 − 2.16 (m, 1H), 1.39 (s, 3H), 1.21 (d, *J* = 9.1 Hz, 1H), 0.83 (s, 3H); ^13^C NMR (101 MHz, CDCl_3_) *δ* 167.2, 159.7, 159.1, 147.4, 146.8, 146.2, 135.8, 130.7, 126.6, 124.6, 119.5, 115.1, 112.3, 55.9, 40.7, 40.3, 37.7, 32.8, 31.2, 29.8, 26.1, 22.6. UPLC, t_R_ = 3.356 min, purity 98.51%.

### Biological evaluation

#### Cell culture and cell viability assay

The U87MG cell line (catalogue iCell-h224) and U251MG cell line (catalogue iCell-h219) were purchased from iCell Bioscience Inc (Shanghai) cell bank. T98G cell line was obtained from Yining Zhang’s group. All cells were cultured in DMEM supplemented with 10% (v/v) FBS and penicillin-streptomycin in a humidified incubator at 37 °C under 5% CO_2_. The different compounds were dissolved in DMSO at the concentration of 10 mM and diluted in serum-free medium. The GBM cells were seeded into 96-well microplates at the density of 6000 cells/well and incubated for 24 h. Fresh media supplemented with different concentrations of the compounds were added, and the cells were cultured for 72 h. The CCK-8 reagent (TargetMol, C0005) was added to each well and the cells were incubated for 4 h at 37 °C. DMSO was added to dissolve the formazan crystals, and the absorbance was measured at 570 nm using a microplate reader (Genios Tecan). An untreated control group was included and its viability was set as 100%. IC_50_ value of the two probes was calculated using GraphPad Prism 5.0.

#### Drug affinity responsive target stability (DARTS) assay

U251 cells were plated in 10 cm dishes. After overnight, the cells were washed by precooled PBS, and lysed by NP-40 lysis buffer for 30 min. Next, lysates were centrifuged at 13000 rpm for 15 min at 4 °C, and protein concentration was determined using a BCA protein assay kit (Solarbio, PC0020). The supernatants were divided into four 50 μL aliquots and incubated with DMSO or compound **C5** (50 and100μM) for 30 min at room temperature. Then, the 5 μL of Pronase (The method of preparing the stock solution is 1 μg of Pronase in 200 μg of lysate, the effect concentration which is diluted by ultrapure water to 200X/400X/600X), and the reaction was stopped by 5× loading buffer. Protein samples were boiled and analysed by using Western blot.

#### Cellular thermal shift assay (CETSA) assay

U251 cells in logarithmic growth were harvested and lysed with protease inhibitors and phosphorylase inhibitors, followed by ultrasonic disruption and ice extraction for 1 h. After that, the lysate was divided into three parts evenly and incubated with DMSO, 50 µM of **C5**, and 100 µM of **C5** for 1 h at room temperature. Subsequently, samples were heated in the PCR machine for 10 min in indicated temperature gradients (40–70 °C), respectively. Finally, samples were centrifuged at 14,000 × g for 30 min at 4 °C to remove aggregates, and the resulting supernatant was analysed with western blotting.

#### Flow cytometry

U251 (1 × 10^5^ cells/well) in logarithmic growth phase were transferred into 6-well microplates and cultured for 12 h. Therefore, U251 cells were treated with **C5** (0, 2.5, 5, and 10 μM) for 24 h. Annexin V-FITC/PI staining kit (Yeasen, Shanghai, China) was used to detect cell apoptosis. The cell cycle distribution was analysed by staining with a cell cycle analysis kit. Intracellular ROS levels were also detected using a ROS assay kit (Yeasen, Shanghai, China). All assays were performed as per the manufacturer’s instructions, and the stained cells were analysed by flow cytometry (Beckman Coulter, Miami, FL) to quantify apoptosis rates, cell cycle distribution, and ROS levels as appropriate.

#### Transwell invasion experiment

Matrigel-coated transwell chambers (24-well; 8-μm pore size, Corning, New York, NY, USA) were used to evaluate the invasion capability of GBM cells. The U251 cells incubated with different doses of **C5** (0, 2.5, 5, and 10 μM) for 24 h were seeded on Matrigel-coated transwell chambers at the density of 2 × 10^4^/well in a serum-free medium. After culturing for 24 h, the cells that invaded through Matrigel to the lower surface of the membrane were fixed with 4% methanol and stained with 0.1% crystal violet. The cells were counted under an inverted microscope in at least 5 random fields per well.

#### Measurement of mitochondrial membrane potential (MMP)

U251 cells were seeded in 6-well microplates at the density of 1.5 × 10^5^ cells/well and cultured overnight. Fresh medium containing different doses of **C5** (0, 2.5, 5, and 10 μM) was added, and the cells were cultured for 24 h. The cells were stained using the JC-1 kit (Sigma-Aldrich, USA) according to the manufacturer’s instructions and observed by fluorescence microscopy. The changes in the ratio of the red and green fluorescence intensities were calculated to evaluate MMP.

#### Western blotting

U251 cells were treated with **C5** (0, 2.5, 5, and 10 μM) for 72h, harvested, and lysed with RIPA buffer containing protease and phosphatase inhibitors. The protein content in the lysates was measured using a BCA protein assay kit, and 20 μg protein per sample was separated by SDS-PAGE and transferred to PVDF membranes (Merck Millipore, Burlington, MA, USA). After blocking with 5% skim milk in Tris buffered saline-Tween (TBS-T) at room temperature for 2h, the blots were incubated overnight with the following primary antibodies at 4 °C: mouse anti-GAPDH (ab8245, 1:10000), rabbit anti-Bax (ab32503, 1:5000), anti-Bcl-2 (ab32124, 1:1000), anti-caspase 3 (ab32351, 1:5000), anti-cleaved caspase 3 (ab32042, 1:500), anti-cleaved PARP-1 (ab32561, 1:1000), anti-LC3B (ab192890, 1:2000), anti-p62 (ab109012, 1:10000), anti-PARP-1 (ab32138, 1:10000). Following three washes with TBS-T, the membranes were incubated with corresponding horseradish peroxidase-conjugated secondary antibodies (1:10000) at room temperature for 90 min, and washed thrice with TBS-T. The bands were visualised using the Minichem@610 Chemiluminescence imager (SAGCEREATION, Beijing) on the chemiluminescence channel.

##### 3D spheroid assay

Malignant glioblastoma tumour cells U251 were seeded in cell-repellent 96-well U-bottom plates (Sbio^™^), and cultured for 72 h to allow spheroid formation. Subsequently, 50 μL of culture medium was removed, and 50 μL of drug-containing medium and incubated for another 72 h. Spheroids were stained with PI and Calcein-AM, and observed under a fluorescence microscope.

#### Molecular docking

Molecular docking was performed using AutoDock Vina^42^,^43^. The crystal structure of human CDK2 in complex with the ligand SU9 (PDB ID: 3PY1) was obtained from the Protein Data Bank (www.pdb.org). The docking site was defined using a grid box with the centre of X = −78.104, Y = −100.511, and Z = 28.775, centred on the centroid of the co-crystallized ligand. The three-dimensional (3D) structure of the test compound was generated using Discovery Studio 3.5. Subsequently, the compound was docked into the ligand-binding site of CDK2 using AutoDock Vina with an exhaustiveness setting of 32 to ensure thorough sampling of possible binding conformations. The resulting docking poses were visualised and analysed using PyMOL (Schrödinger, LLC).

## Supplementary Material

Supporting Information_revised_no author.docx

## Data Availability

The authors confirm that the data supporting the findings of this study are available within the article and its Supplementary Materials.
