# Supplementary material for: Design, synthesis, and evaluation of novel pinane-based thiazolidione derivatives with anti-glioblastoma activity
Source: J Enzyme Inhib Med Chem. 2025 Sep 4;40(1):2553691. doi: 10.1080/14756366.2025.2553691 (PMC12412324; doi:10.1080/14756366.2025.2553691)
Supplement: Supporting Information_revised_no author.docx [file IENZ_A_2553691_SM1506.docx]

Supporting Information

**Design, Synthesis, and Evaluation of Novel Pinane-Based Thiazolidione Derivatives with Anti-glioblastoma Effects**

**Figure S1** ^1^H NMR spectrum (CDCl_3_; 400MHz) of **C1**

**Figure S2** ^13^C NMR spectrum (CDCl_3_; 101MHz) of **C1**


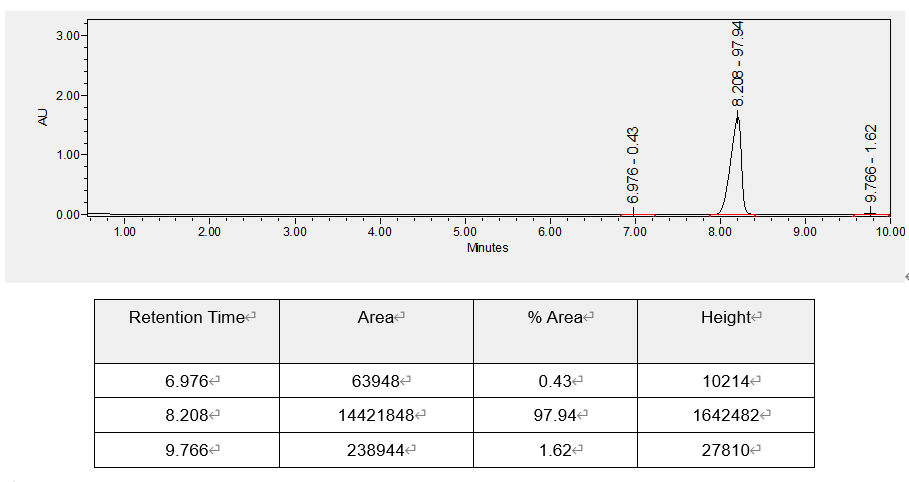


**Figure S3** UPLC spectrum of **C1**

**Figure S4** ^1^H NMR spectrum (CDCl_3_; 400MHz) of **C2**

**Figure S5** ^13^C NMR spectrum (CDCl_3_; 101MHz) of **C2**


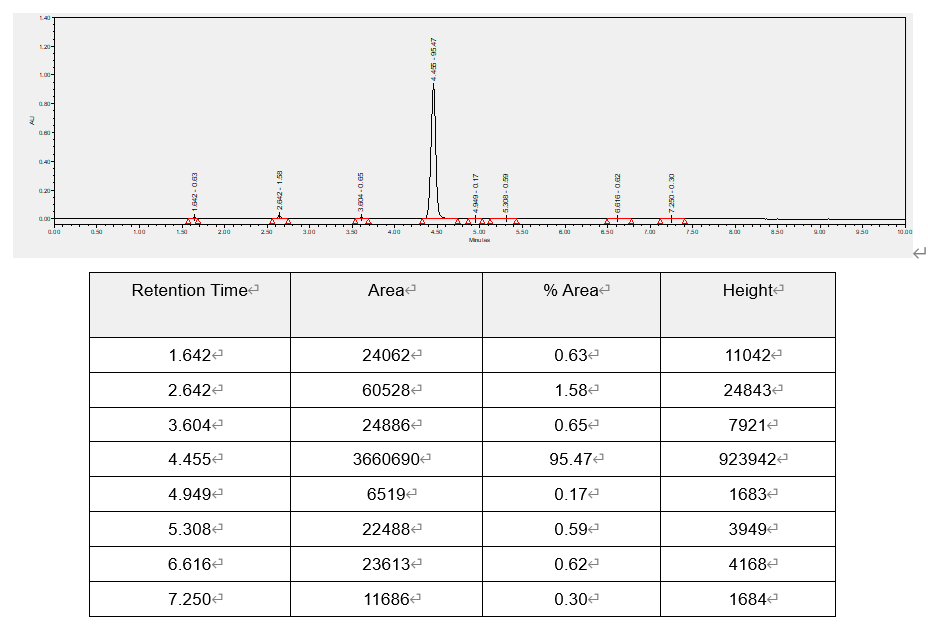


**Figure S6** UPLC spectrum of **C2**

**Figure S7** ^1^H NMR spectrum (CDCl_3_; 400MHz) of **C3**

**Figure S8** ^13^C NMR spectrum (CDCl_3_; 101MHz) of **C3**


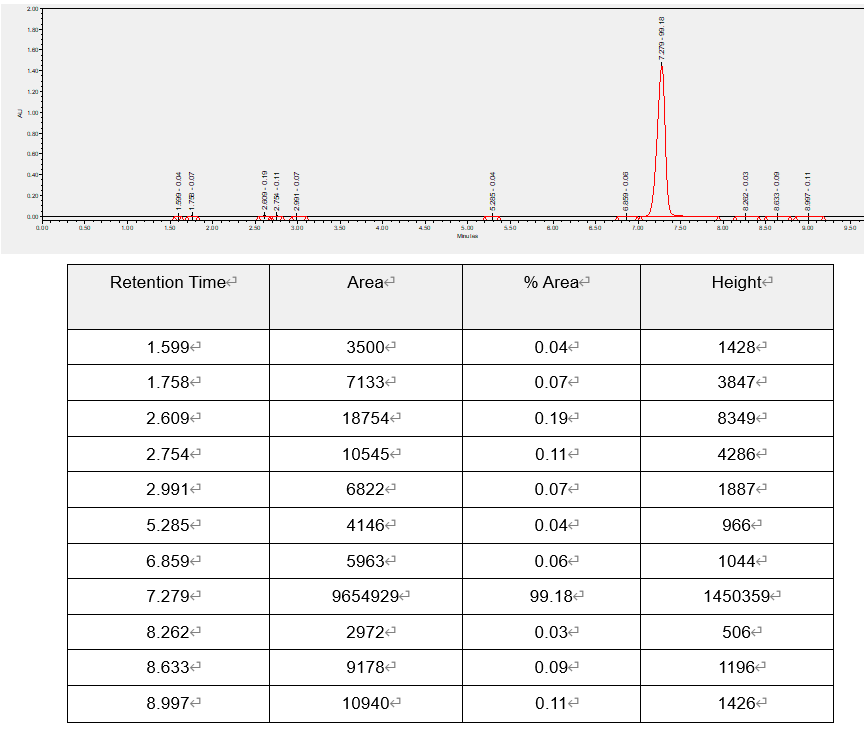


**Figure S9** UPLC spectrum of **C3**

**Figure S10** ^1^H NMR spectrum (CDCl_3_; 400MHz) of **C4**

**Figure S11** ^13^C NMR spectrum (CDCl_3_; 101MHz) of **C4**


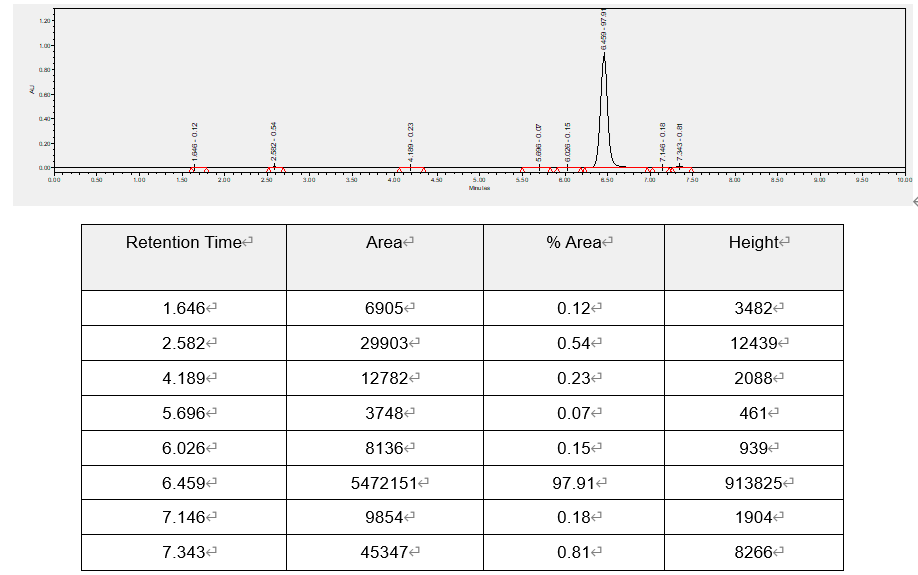


**Figure S12** UPLC spectrum of **C4**

**Figure S13** ^1^H NMR spectrum (CDCl_3_; 400MHz) of **C5**

**Figure S14** ^13^C NMR spectrum (CDCl_3_; 101MHz) of **C5**


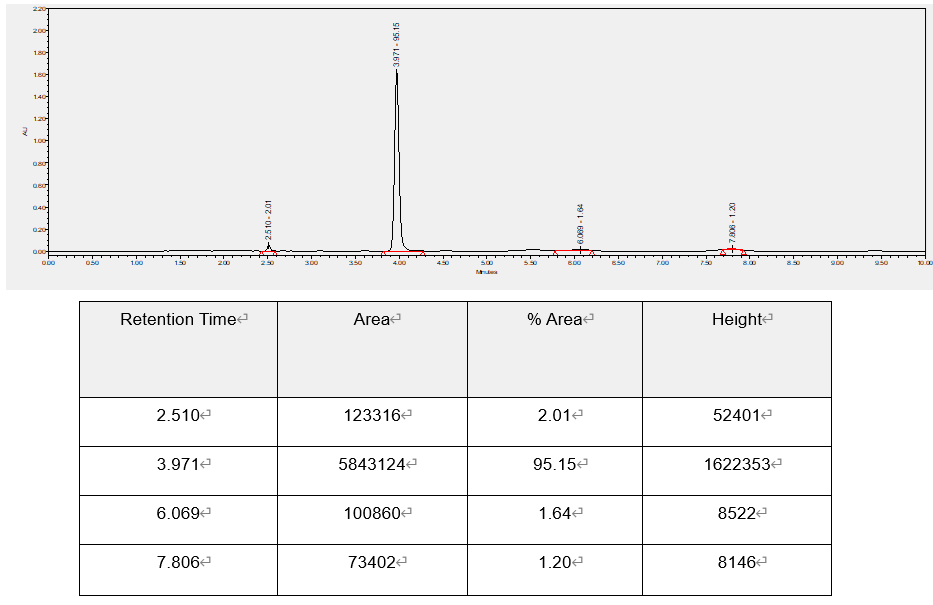


**Figure S15** UPLC spectrum of **C5**

**Figure S16** ^1^H NMR spectrum (DMSO-*d_6_*; 400MHz) of **C6**

**Figure S17** ^13^C NMR spectrum (DMSO-*d_6_*; 101MHz) of **C6**


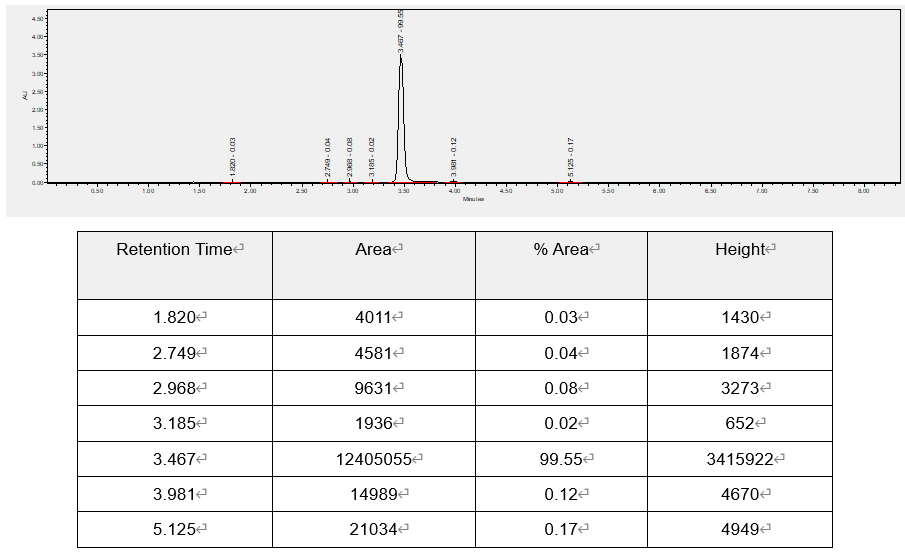


**Figure S18** UPLC spectrum of **C6**

**Figure S19** ^1^H NMR spectrum (CDCl_3_; 400MHz) of **C7**

**Figure S20** ^13^C NMR spectrum (CDCl_3_; 101MHz) of **C7**


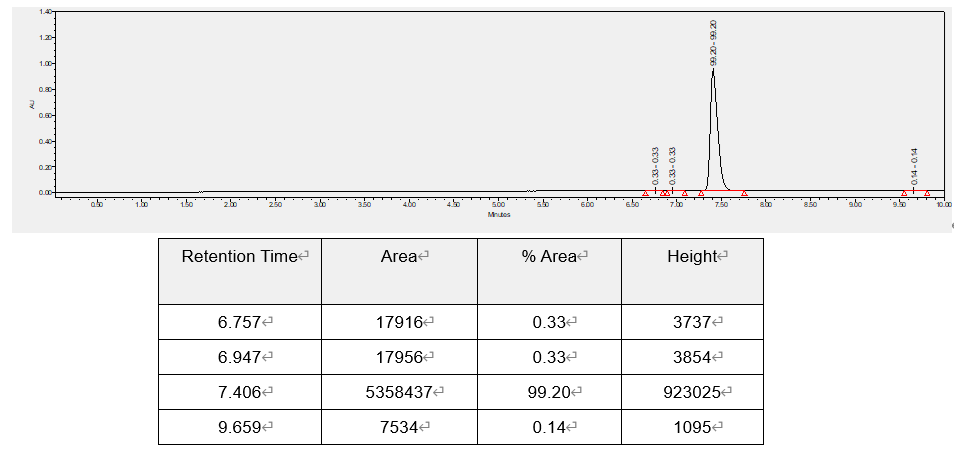


**Figure S21** UPLC spectrum of **C7**

**Figure S22** ^1^H NMR spectrum (Pyridine-*d_5_*; 400MHz) of **C8**

**Figure S23** ^13^C NMR spectrum (Pyridine-*d_5_*; 101MHz) of **C8**


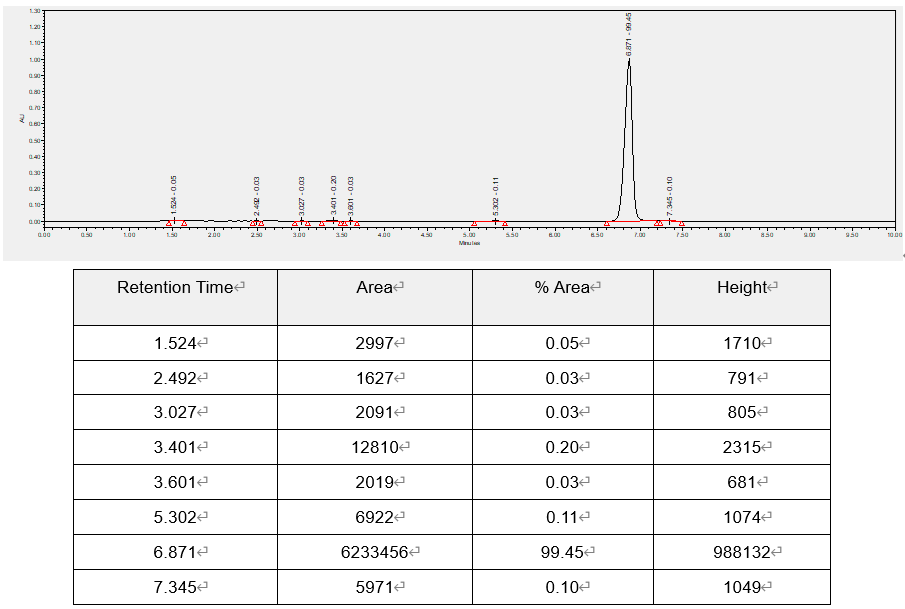


**Figure S24** UPLC spectrum of **C8**

**Figure S25** ^1^H NMR spectrum (DMSO-*d_6_*; 400MHz) of **C9**

**Figure S26** ^13^C NMR spectrum (DMSO-*d_6_*; 101MHz) of **C9**


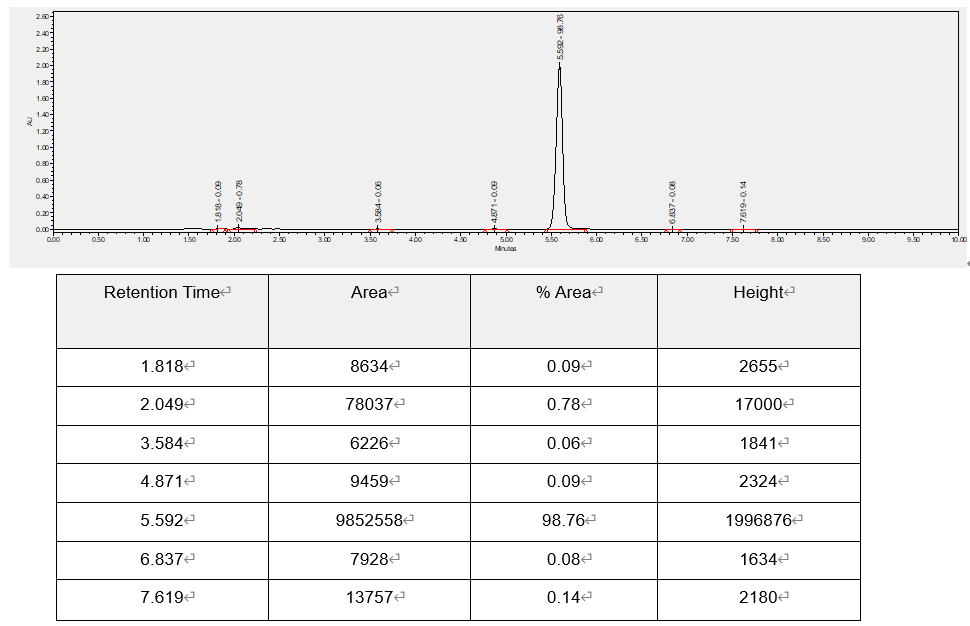


**Figure S27** UPLC spectrum of **C9**

**Figure S28** ^1^H NMR spectrum (CDCl_3_; 400MHz) of **C10**

**Figure S29** ^13^C NMR spectrum (CDCl_3_; 101MHz) of **C10**


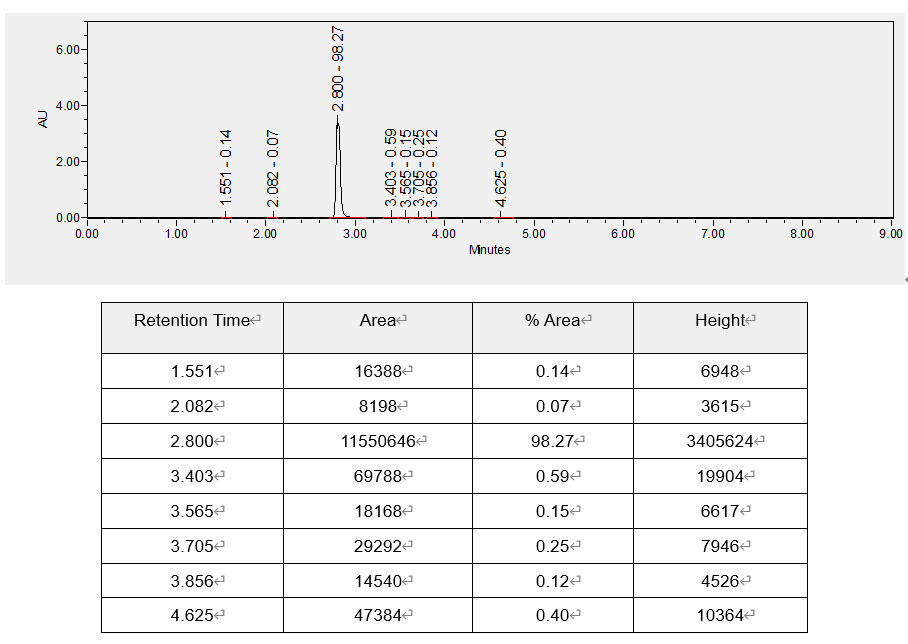


**Figure S30** UPLC spectrum of **C10**

**Figure S31** ^1^H NMR spectrum (DMSO-*d_6_*; 400MHz) of **C11**

**Figure S32** ^13^C NMR spectrum (DMSO-*d_6_*; 101MHz) of **C11**


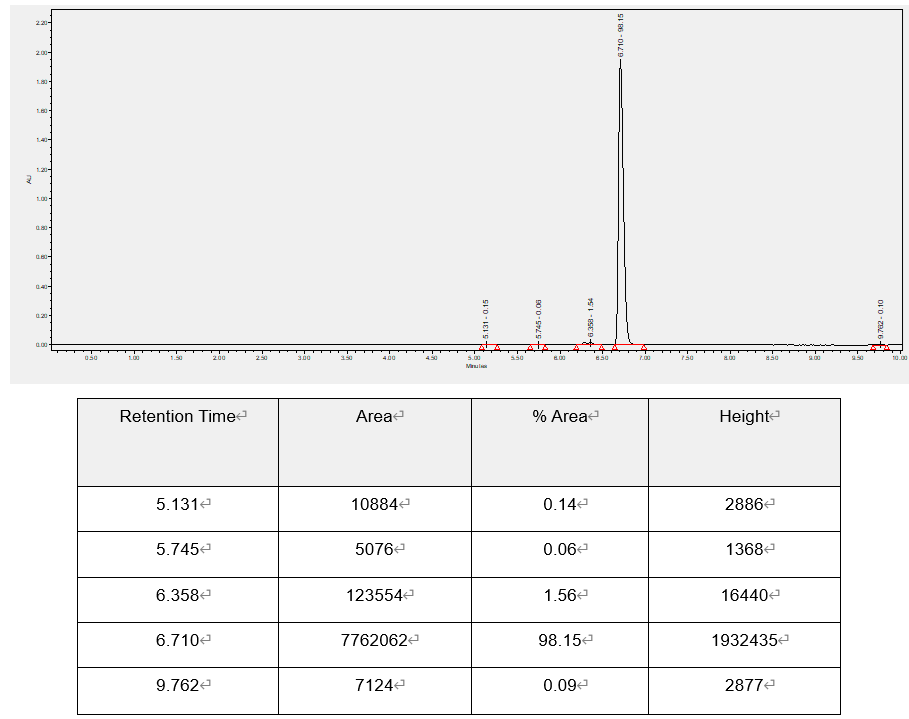


**Figure S33** UPLC spectrum of **C11**

**Figure S34** ^1^H NMR spectrum (DMSO-*d_6_*; 400MHz) of **C12**

**Figure S35** ^13^C NMR spectrum (DMSO-*d_6_*; 101MHz) of **C12**


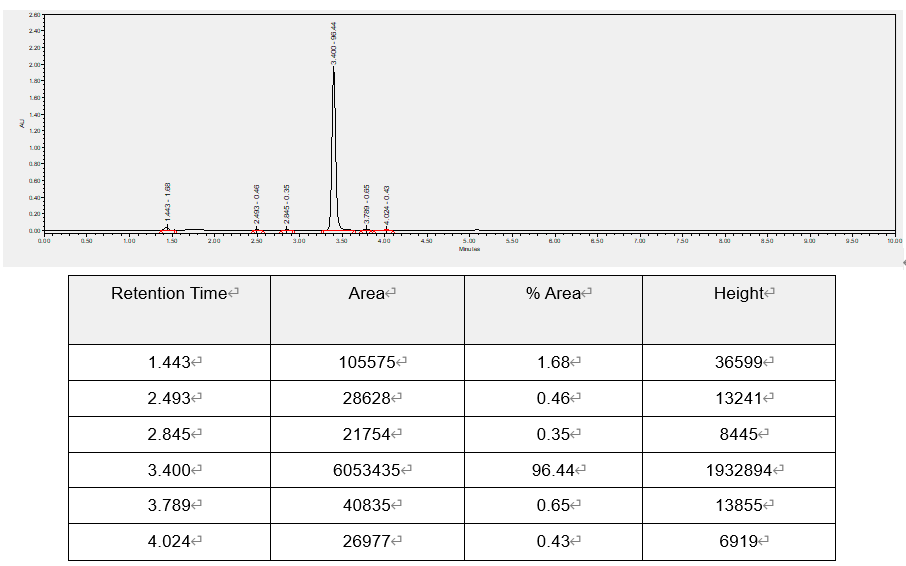


**Figure S36** UPLC spectrum of **C12**

**Figure S37** ^1^H NMR spectrum (DMSO-*d_6_*; 400MHz) of **C13**

**Figure S38** ^13^C NMR spectrum (DMSO-*d_6_*; 101MHz) of **C13**


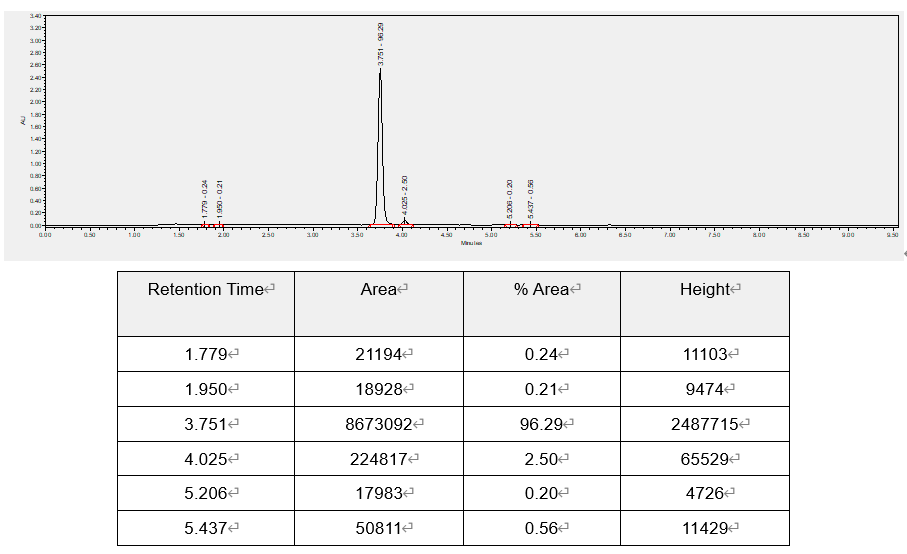


**Figure S39** UPLC spectrum of **C13**

**Figure S40** ^1^H NMR spectrum (CDCl_3_; 400MHz) of **C14**

**Figure S41** ^13^C NMR spectrum (CDCl_3_; 101MHz) of **C14**


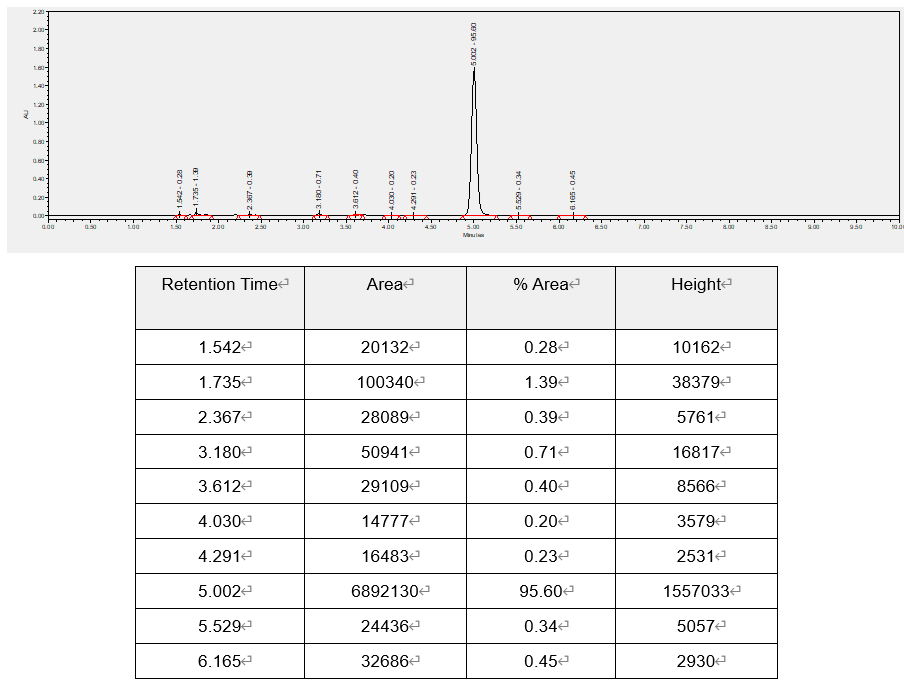


**Figure S42** UPLC spectrum of **C14**

**Figure S43** ^1^H NMR spectrum (CDCl_3_; 400MHz) of **C15**

**Figure S44** ^13^C NMR spectrum (CDCl_3_; 400MHz) of **C15**


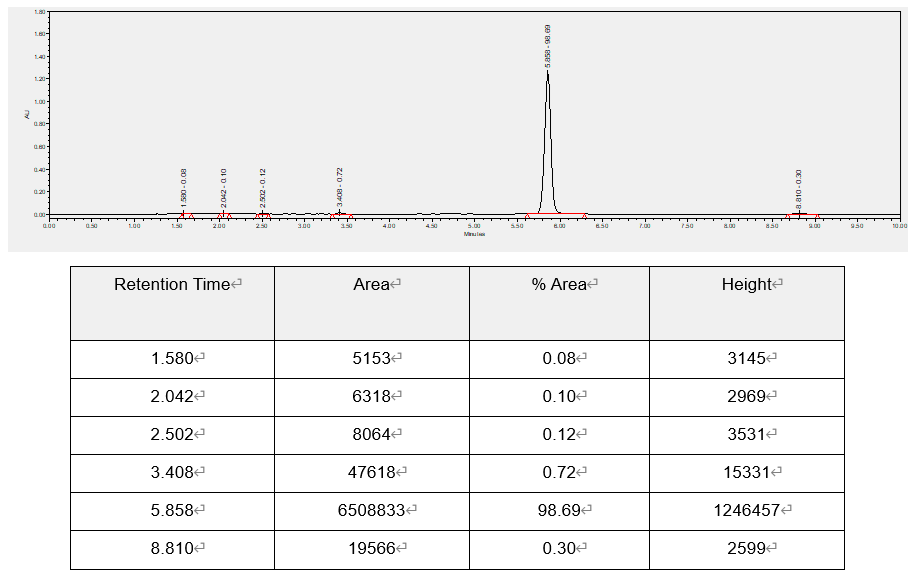


**Figure S45** UPLC spectrum of **C15**

**Figure S46** ^1^H NMR spectrum (DMSO-*d_6_*; 400MHz) of **C16**

**Figure S47** ^13^C NMR spectrum (DMSO-*d_6_*; 400MHz) of **C16**


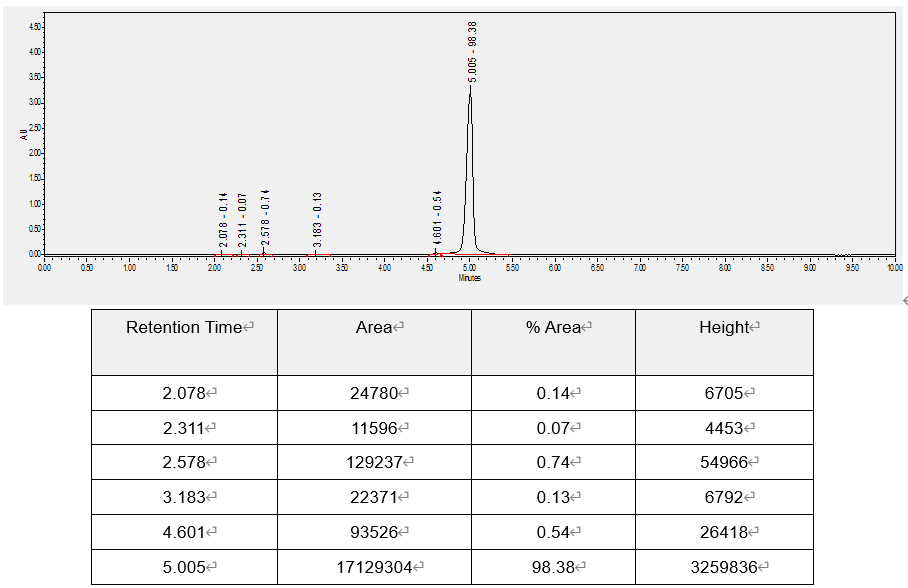


**Figure S48** UPLC spectrum of **C16**

**Figure S49** ^1^H NMR spectrum (CDCl_3_; 400MHz) of **C17**

**Figure S50** ^13^C NMR spectrum (CDCl_3_; 400MHz) of **C17**


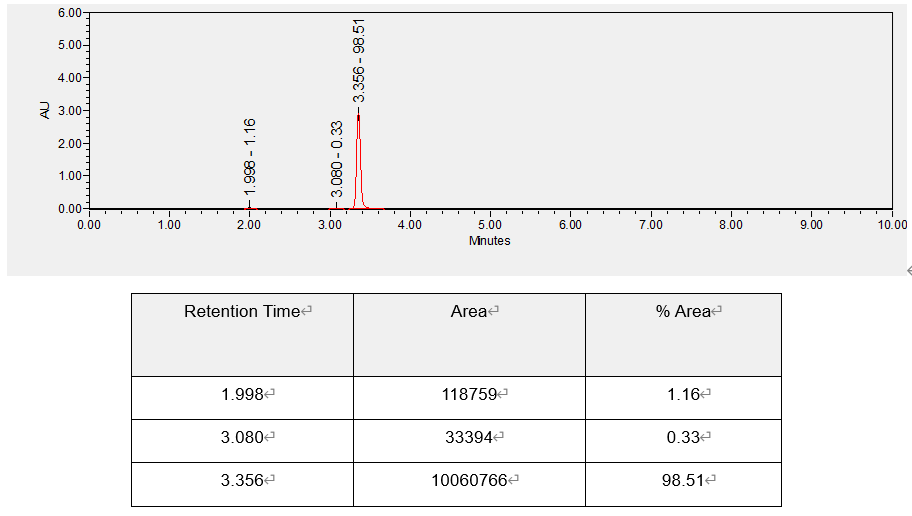


**Figure S51** UPLC spectrum of **C17**
